# Protecting Mental Health Data Privacy in India: The Case of Data Linkage With Aadhaar

**DOI:** 10.9745/GHSP-D-20-00346

**Published:** 2021-09-30

**Authors:** Ameya Bondre, Soumitra Pathare, John A. Naslund

**Affiliations:** a Digital Mental Health Research Consultant, Mumbai, India.; b Centre for Mental Health Law and Policy, Indian Law Society, Pune, India.; c Department of Global Health and Social Medicine, Harvard Medical School, Boston, MA, USA.

## Abstract

In an underprepared and under-resourced digital mental health system, the linkage of health and personal data with Aadhaar, a biometric system that provides a unique identification number to all Indian residents, poses significant privacy risks to individuals seeking mental health care. We discuss the challenges in protecting mental health data privacy due to these emerging digital health technologies.

## Key Messages

- Under the Aadhaar system, biometric and demographic data stored in a central database can pose a significant threat to the data privacy of individuals with potentially stigmatizing conditions such as mental health disorders.
- The emerging use of artificial intelligence in digital solutions (including health interventions) can further complicate this situation. There is often patient exclusion in the development of artificial intelligence systems in mental health research and clinical practice.
- Based on Global Data Protection Regulation and other data privacy regulations, this article provides guidelines for mental health policy makers, professionals, technologists, and related health system stakeholders to protect the individual’s data privacy.

## INTRODUCTION

The Mental Health Care Act 2017 in India represents a landmark legislation advocating for the rights, dignity, and autonomy of persons facing the challenges of mental illness and aims to transform the delivery of mental health care across the country.^1^^,^^2^ The new law mentions digital data privacy; yet few studies have focused on this to date.^3^ This has contributed to its low prioritization in emerging digital mental health programs in India.

The Government of India has made a systematic effort to ensure that all health service clients have a unique health identity (UHID), a digital identity issued by health care providers to track patients and secure relevant health documents, and link the UHID to the unique identification number assigned to every Indian resident, called the “*Aadhaar*” number.^4^^,^^5^ This linkage raises critical questions of how well the system and the community-at-large are prepared for such a large-scale data linkage and its implications for privacy. This has especially important implications for individuals living with mental illness, as safeguarding their data privacy is essential to reduce their risk of being judged or facing stigma, hostility, or adversities in personal or workplace relationships.

In this commentary, we discuss the challenges in protecting mental health data privacy, guidelines to protect the personal data privacy of individuals with mental health disorders in India, and implications for digital mental health services in other low-resource settings.

## BENEFITS AND RISKS OF DIGITAL DATA SHARING

Internet penetration in India has shown consistent growth in adoption in urban and rural areas,^6^ which has brought about increasing interest in digital tools for various aspects of health care. This includes mobile-based services for providing health information^7^^,^^8^ and mobile phone reminders for offering education or counseling in the context of HIV,^9^^,^^10^ diabetes,^11^ TB,^12^ and cardiovascular diseases.^13^^,^^14^ There is also mounting interest in digital resources for mental health care, reflected in recent analyses of short message service (SMS)-based services^15^^,^^16^ for mental health issues, with SMS and voice reminders used to reduce missed appointments and improve follow-up at an urban community mental health clinic^15^; and use of tele-helplines for crisis resolution and follow-up.^16^ Use of artificial intelligence (AI) has also been reported in the case of commercial smartphone applications in India that are freely accessible to users.^17^^,^^18^ One such example is “Wysa,” an AI-enabled, empathetic, text-based conversational mobile mental well-being app, that has shown improvement in the mood of users with self-reported symptoms of depression.^17^

Importantly, the Government of India has emphasized the need to scale up digital mental health solutions due to the significant gap between those in need of care and those who receive mental health care, referred to as the “treatment gap.”^19^^,^^20^ It is estimated that 90% of the roughly 200 million people in India who live with mental health disorders^21^ lack access to necessary services^22^; yet many of these individuals own smartphones (as phone users represent 88.5% of people in India and more than 625 million internet subscribers^23^). The National Mental Health Survey (2016) also recommended an expanded usage of smartphone-based applications, digital tools for decision-support (due to the scarce number of psychiatrists), and electronic databases for follow-up of individuals with mental health disorders.^24^^,^^25^ This would enable large-scale mental health data sharing between the heterogeneous providers (i.e., specialists, primary care doctors, frontline workers, informal healers), patients, and other stakeholders.^26^ Among the existing studies that have evaluated digital mental health services in India,^3^^,^^15^^,^^16^^,^^27^ there has been limited focus on data privacy.^27^ With the increase in digital data sharing on clinical, demographic, occupational, and social variables, this potentially raises individual privacy concerns.

The Government of India has emphasized the need to scale up digital mental health solutions due to the significant gap between those in need of care and those who receive mental health care.

Furthermore, there is significant social stigma surrounding mental health conditions, which impedes individual care seeking, social participation, and access to treatment.^28^ With the widespread challenges in overcoming stigma and negative attitudes toward mental health conditions,^29^^,^^30^ it is critical to safeguard the privacy and confidentiality of users’ mental health data, especially as they interface with digital health systems. Stigma is negatively correlated with help seeking for allopathic or modern medical treatment in the Indian context, while a positive association has been shown with previous informal help seeking.^28^^,^^31^ Stigma motivates families to conceal the affected person, often hiding the condition and its perceived causes (driven by shame) such as previous sins or bad acts, which can substantially delay or inhibit timely access to treatment.^28^^,^^31^ Therefore, protecting the data privacy of individuals with potentially stigmatizing mental health disorders is critical as unintended disclosure could impede their access to care, result in possible denial of additional services, or result in possible discrimination by employers or agencies providing financial aid for treatments.

## THE AADHAAR SYSTEM AND ITS LINKAGES

Under the Government of India’s Ministry of Electronics and Information Technology, the Aadhaar is a 12-digit unique number assigned to every Indian resident to record demographic (name, address, date of birth, and sex) and biometric data (fingerprints, iris scans, and a photograph). Aadhaar identification helps deliver subsidies, cash benefits, and incentives to intended beneficiaries, but the number has been increasingly linked to bank and income tax accounts, mobile phone numbers, and social welfare programs such as disability and elderly pension schemes.^32^^,^^33^ This is pertinent in the context of seeking treatment for stigmatizing mental health conditions, where accessing care will be tied to compulsory linking of personal identification information (i.e., Aadhaar).

### Health Consequences of Linking Data

The consequences of poorly regulated data linkage have begun to show. In 2017, the Government issued a notification to mandatorily link the Aadhaar number with the patient identification number for patients with TB to receive cash assistance under the Revised National TB Control Program. This led to an interruption in treatments, particularly in cases of patients from lower socioeconomic segments, due to the documents and procedures required for availing an Aadhaar number.^34^ There have been instances of patients with HIV and AIDS dropping out of antiretroviral therapy, fearing a breach of privacy, when it was made compulsory to include Aadhaar numbers in their treatment reports.^35^ It should be noted that similar to mental health disorders, TB and AIDS carry a considerable social stigma in India.

The consequences of poorly regulated data linkage have begun to show.

A breach of privacy leading to the denial of a health service to an individual also leads to loss of their autonomy (when benefits are denied and there is no alternative mode of identification that is permitted) and loss of dignity (compromise of the individual’s right to physical or mental integrity, as confidential data are leaked without consent). Both of these losses can potentially worsen the situation for individuals with a mental illness and their families.^36^ Moreover, being identified as having a mental health problem in India can lead to institutionalized discrimination and loss of civil rights; for example, the loss of a job,^19^^,^^37^ denial of the right to vote,^38^ divorce on grounds of mental illness (under the Hindu Marriage Act),^39^ and automatic questioning of an individual’s capacity to make a will.^40^

### Unique Challenges of the Aadhaar Data Linkage

Poor regulation of data linkage has other grave consequences such as systemic leakages, as illustrated by the case of about 200 government websites that inadvertently displayed the Aadhaar numbers of individuals^41^ and technologists now working for for-profit companies, who were previously involved in the formulation of the Aadhaar system, in the absence of strict regulations to prevent conflict of interest.^42^ It is not uncommon for health systems to adopt more integrated digital infrastructures, requiring the implementation of new protections for the privacy of users. However, in the case of the Aadhaar system, there are unique challenges and serious threats to privacy,^43^ as described in the following points.

1. Other laws in India such as the Registration Act (concerning the mandatory registration of documents of Indian citizens), collect biometric information, as with the Aadhaar system. However, such usage of biometric data comes with stringent legal restrictions specified in the Act,^43^ adhering to the principle of “purpose limitation,” (or processing of personal data for specified, explicit, and legitimate purposes only; further processing shall not be incompatible with initial purposes). These restrictions have not been mentioned in the Aadhaar Act of 2016.^33^
2. Under the Aadhaar system, biometric and demographic data are stored in a centralized database and associated with the individual’s unique Aadhaar number. This number is sought to be “seeded” (added as a new data field) with other public and private databases in the country.^43^ Normally, we have access to our different data “buckets” (e.g., details on air travel, bank accounts, mobile phones, employment histories, or health records), and only we can construct our full “profile” through these separate data buckets. But if the Aadhaar number is seeded into databases, which to some extent has already begun via linkage of Aadhaar numbers with bank accounts and mobile phone numbers, then these data buckets will become integrated. Therefore, individuals lose control over who can reconstruct their profile. There is a serious concern reported that potentially unauthorized persons in the government would then be able to “profile” an individual by pulling out information from various databases using the Aadhaar number.^43^ This has other implications too, such as self-censorship and the likely suppression of dissent or public opinion sharing in democratic systems of governance.^44^
3. Aadhaar proponents claim that this system allows us to “see individual lives in different spheres”^43^ to conduct big data analysis, such as econometric and epidemiological analyses, and thus, discover hidden data patterns to establish predictive and/or causal relationships between multiple domains of the economy. However, this very “personal data economy”^43^^,^^45^ could potentially monetize information about individuals’ private lives, much before the creation of sufficient digital literacy or safeguards.
4. While we have become aware that smartphones, social media platforms, or Internet search engines may violate our privacy, technologies such as encryption or virtual private networks can protect user privacy to an extent. Aadhaar’s centralized system of data integration lacks these safeguards.^43^
5. The safeguards against data breaches in the 2016 Aadhaar Act warrant greater scrutiny and strengthening. For example, if data are “leaked,” only the Unique Identification Authority of India^32^^,^^33^—not the affected person—is authorized to file a First Investigation Report, which invests the power to prosecute in the government agency and not the individual whose privacy has been violated.

### Broken Consent Mechanism

The Aadhaar system suffers from a “broken consent mechanism” as best illustrated in the recent case of registration of Indian citizens on the Government’s CoWin vaccine portal for COVID-19 vaccination.^46^ While the government has reiterated that Aadhaar is not mandatory for vaccine registration and that any identity proof would be accepted for vaccination, the realities are playing out differently. The Government’s operational guidelines encourage vaccine officers to verify the recipient’s identity with Aadhaar ID, compared to other forms of identification. In other words, Aadhaar is the “preferred mode” for authentication, and although described as “voluntary,” it is being made “mandatory” for all practical purposes, as in the case of other services such as linkage with bank accounts or registration for mobile phones.

Aadhaar suffers from a “broken consent mechanism” as best illustrated in the recent case of registration of Indian citizens on the Government’s CoWin vaccine portal for COVID-19 vaccination.

### Data Erasure

Finally, the Aadhaar system suffers from an absence of the facility of data erasure offered to the data subject or user,^33^ as enshrined in data protection and privacy laws in other regions globally such as the General Data Protection Regulation (GDPR) in the European Union (further detailed in the next section). This means an absence of the user’s “right to be forgotten” where the data subject has the right to the erasure of personal data concerning themselves without undue delay on certain grounds as mentioned in Article 17 of the GDPR.^47^ Some examples of such grounds for data erasure include the subject withdrawing consent or opposing the processing of their personal data, unlawful processing of data, or the personal data being no longer necessary in relation to the purposes for which they were originally collected or processed.

## FRAMEWORKS FOR PROTECTING PERSONAL DATA PRIVACY

There are key international frameworks and methodologies aimed at protecting personal data privacy. These can inform the development of similar frameworks for the Indian context or incorporate key features into existing Indian policy, legal, and/or ethical frameworks.

### General Data Protection Regulation

The GDPR, which came into force in May 2018, is a case in point. Although GDPR guidelines apply to organizations in the EU, they have important privacy considerations that are generalizable. GDPR encourages the development of digital systems that are less privacy invasive. The GDPR defines data pertaining to health as^48^:

*Personal data related to the physical or mental health of a natural person, including the provision of health care services, which reveal information about his or her health status.*

GDPR also describes “genetic data” as the characteristics of a natural person that give unique information about her physiology or health, and “biometric data” as information obtained from a specific technical processing relating to the physical, physiological, or behavioral characteristics of a natural person, which allow or confirm their unique identification.

The processing of these kinds of personal health data is prohibited unless the subject has given “explicit consent” or if the processing of such data is necessary for preventive or occupational medical care (e.g., assessment of an employee’s working capacity, medical diagnosis, provision of health care, or social benefits), for reasons of public health interest such as protecting against serious cross-border threats to health, or ensuring optimum standards of quality and safety of health care products or services. Table 1 details the principles of the GDPR.^49^

**TABLE 1.** Principles of the GDPR Guidelines From the European Union

Principle	Description
1. Lawfulness, fairness, and transparency	Transparent processing of personal data in relation to the subject.
2. Purpose limitation	Processing of personal data for specified, explicit, and legitimate purposes only; further processing for archiving in the public interest, or for scientific/historical/statistical research (according to Article 89[1] of GDPR) shall not be incompatible with the initial purposes.
3. Data minimization	Personal data should be adequate, relevant, and limited in relation to the purpose of processing.
4. Accuracy	Personal data should be accurate and up-to-date; inaccurate data should be erased or rectified without delay and regarding the purposes for which they are processed.
5. Storage limitation	Personal data are to be kept in a form that permits identification of subjects for no longer than is necessary for the purposes for which their data are processed; personal data may be stored for longer periods for archiving in the public interest, or for scientific/historical/statistical research (according to Article 89[1] as above), subject to the technical and organizational measures required by this regulation.
6. Integrity and confidentiality	Personal data are to be processed to ensure their appropriate security, including protection against unauthorized or unlawful processing, accidental loss, destruction, or damage, using appropriate technical or organizational measures.
7. Accountability	The “controller” (for example, the project head or signing authority of the project) shall be responsible for, and be able to demonstrate compliance to the aforesaid principles.

Abbreviation: GDPR, General Data Protection Regulation.

Under the GDPR, organizations must conduct a Data Protection Impact Assessment (DPIA)^50^ that includes documentation of the need to conduct such an assessment, a detailed explanation of data processing, the data controller’s (e.g., the project head) consultation with relevant stakeholders, compliance and proportionality measures undertaken in the project, and a description of likely data privacy risks, their potential impact on individuals, and steps taken to mitigate/eliminate these risks. Table 2 includes a summary of the DPIA requirements.

**TABLE 2.** DPIA Checklist (points for documentation) to be Followed by Organizations Who Are Bound by the GDPR Guidelines

Section	Description
1. The need for a DPIA	The aims of the project; types of data processing involved; and the reasons to identify the need for a DPIA
2. Data processing	Nature: method of collection, usage, storage, and deletion of data; source of data; details on sharing of data with anyone; any likelihood of high-risk data processing
	Scope: nature of data, any inclusion of special category or criminal offense data, sample size and data collection frequency, duration of data storage, scope of geographical area and individuals affected
	Context: nature of the relationship between the data controller [for example, the project head] and the individual, degree of control exercised by the individuals on their data, individuals’ expectations on the usage of their data, any data on children or vulnerable groups, prior concerns or security flaws or current public concerns related to the data processing, novelty of data processing, current state of technology around data processing, and whether the controller has signed up for any approved code of conduct
	Purposes: aim of the project, intended effects on individuals, benefits of data processing for the controller, and broader benefits
3. Controller’s consultation with stakeholders	The controller’s consultation process with relevant stakeholders; the need and timing of seeking individuals’ views on their data; the details of project collaborating partners; any consultations planned with information security or other kinds of experts
4. Compliance and proportionality measures	Lawful basis for data processing; justification of its purpose; alternate ways of achieving project aims; steps to ensure data quality and data minimization; nature of information provided to the individuals and ways to support their rights; ways to ensure that data processors and analysts comply with all stated steps; methods of safeguarding domestic and international data transfers (if any)
5. Privacy risks and their impact	The source(s) of potential data privacy risk and nature of their potential impact on the individual
6. Mitigation	Measures taken to reduce or eliminate the privacy risks

Abbreviations: DPIA, Data Protection Impact Assessment; GDPR, General Data Protection Regulation.

### Global Initiative on Ethics of Autonomous and Intelligent Systems

The Institute of Electrical and Electronics Engineers (IEEE) Global Initiative on Ethics of Autonomous and Intelligent Systems addresses ethical issues raised by the development and dissemination of new digital systems, which is especially relevant to emerging ways of obtaining digital health data.^51^ This initiative offers guiding principles of “ethically aligned design” (Table 3^52^) and has identified more than 120 key ethical issues and provided recommendations to address them. Currently, the IEEE standards association is developing “standardization projects” to guide technologists and organizations to mitigate the chances of ethical violations of personal data privacy.^51^

**TABLE 3.** General Principles of Ethically Aligned Design of A/IS Outlined by the IEEE Global Initiative on Ethics of Autonomous and Intelligent Systems

#	Principle	Description
1	Human rights	A/IS should be created and operated to respect, promote, and protect internationally recognized human rights.
2	Well-being	A/IS creators should adopt increased human well-being as a primary development criterion.
3	Data agency	A/IS creators should empower individuals with the ability to access and securely share their data (thus, control their identity).
4	Effectiveness	A/IS creators should provide evidence of the system’s effectiveness and fitness for its intended purpose.
5	Transparency	The basis of a particular A/IS decision should always be discoverable.
6	Accountability	A/IS should be developed for providing an unambiguous rationale for all decisions made.
7	Awareness of misuse	A/IS creators should guard against all potential misuses/risks of A/IS in action.
8	Competence	A/IS creators should specify and operators should adhere to the knowledge and skill required for safe and effective operation.

Abbreviations: AIS, autonomous and intelligent system; IEEE, Institute of Electrical and Electronics Engineers.

### Applying Frameworks to Protect Mental Health-Related Data

There is an immediate need to consider the data protections outlined in the GDPR, DPIA, and IEEE Global Initiative on Ethics of Autonomous and Intelligent Systems given the rising interest in digital mental health technologies in India^53^ and resulting personal data sharing at scale. Moreover, the absence of an existing Indian framework on mental health data privacy (except for the clauses in the Mental Health Care Act) has generated limited knowledge on data privacy risks for individuals living with mental health conditions, which faces additional threats posed by the comprehensive Aadhaar linkage spanning individuals’ personal data domains.

The absence of an existing Indian framework on mental health data privacy has generated limited knowledge on data privacy risks for individuals living with mental health conditions.

## ARTIFICIAL INTELLIGENCE AND PRIVACY IN MENTAL HEALTH

Artificial intelligence has begun to penetrate digital mental health solutions, driven in part by the National Strategy on Artificial Intelligence released by the Government of India.^54^ Digital interventions allow opportunities for immense data collection, and AI systems using mathematical algorithms^55^ can seek to make sense of these complex and vast datasets.^56^ The use of AI has been reported in certain algorithm-based mental health applications^17^^,^^18^; however, such an intervention ecosystem has a fundamental contradiction to the importance of consent and data minimization, as articulated in Indian data protection frameworks such as the Sri Krishna report.^57^ Linking Aadhaar can make such systems more invasive by obtaining far greater amounts of personal data from individuals. Mental health data points vary due to the context and characteristics of the individual and the disorder, which can complicate the correlations made by AI systems. In addition, meaningful consent is already hard to achieve in the majority of clinical settings in India due to low awareness, literacy, and agency to exercise the right to informed choice; and therefore, consent can get further complicated if clinical data are automatically fed into an AI system. In these situations, it will be difficult for individuals living with mental health conditions to interpret and/or exercise consent, or for their family members, because data are often correlated in ways that are not identifiable, or where the impacts are not immediately known.^56^

AI algorithms have several other complex applications, notably, predictive modeling.^58^ Broadly, predictive modeling leverages large quantities of personal data to uncover patterns to predict future health outcomes, which could inform treatment selection and treatment personalization.^59^ However, this approach fails to recognize the central role of the patients, especially when their personal data will be used for developing such algorithms.^58^ Consequently, the mental health patient is not sufficiently mentioned as a central collaborator, or the final beneficiary to whom both clinicians and data scientists are accountable.^60^ These challenges related to the use of AI in mental health research and practice demand far greater scrutiny and effort on the part of regulators and policy makers to safeguard the personal data privacy of individuals with mental health conditions.

Provision of the Aadhaar number by an individual having a mental health condition or by his/her family member should be made completely voluntary.

Efforts are equally needed by AI researchers to bridge the gaps in data and technology literacy for both patients and clinicians.

## RECOMMENDATIONS TO SAFEGUARD MENTAL HEALTH PRIVACY

The Government of India’s policy think-tank, NITI Aayog, published a discussion paper on the National Strategy on Artificial Intelligence having guidelines concerning privacy issues in India.^54^ In the absence of specific guidelines for the mental health context, we refer to NITI Aayog’s guidelines to draft customized recommendations for safeguarding the data privacy of individuals in India with mental health conditions. The following 10 measures can be considered by mental health policy makers, professionals, technologists, and related health system stakeholders to protect the individual’s data privacy, in the context of increasing access to and use of digital interventions for mental health.

1. Organizations working in the mental health space should adhere to the core principles of data protection such as informed consent and “data minimization” (i.e., personal mental health data should be adequate, relevant, and limited to the purpose of data collection). This should be supported by data-protection laws that are flexible to include changing technologies, relevant in mental health where a range of digital interventions are being piloted in low-income or middle-income countries or “technology agnosticism.”^53^
2. Provision of the Aadhaar number by an individual having a mental health condition or by his/her family member should be made completely voluntary and not encouraged by the care provider, staff member, or anyone else in the health system interfacing with the individual. The number should be de-linked from the provision of service or any information related to the service. We frame this recommendation based on the Supreme Court of India’s 2018 decree that Aadhaar is not mandatory^61^ and the preceding Supreme Court 2017 judgment protecting the Right to Privacy, as an intrinsic part of the Right to Life and Personal Liberty as guaranteed under the Indian Constitution. In the 2017 judgment, 3 distinct connotations of individual privacy were defined^62^: (1) “spatial control” or creation of private spaces; (2) “decisional autonomy” or intimate choices such as those governing reproduction, faith, or modes of dress; and (3) “informational control,” or use of privacy as a shield to retain control over personal information.
3. Organizations in digital and traditional mental health systems seeking personal data (including passwords, financial data, and biometric information) should maintain reasonable security to protect sensitive personal data and should be held liable for damages when their negligence results in wrongful loss or harm to any person. In India, this aligns with Section 43A of the Information Technology (IT) Act 2000.^63^^,^^64^ The act was amended in 2011 to frame the “IT Rules”^63^ (Table 4), which should be upheld at all levels of a mental health system. Rule 3 of these “2011 IT Rules” includes the following as “sensitive personal data”: information relating to passwords, credit or debit cards, biometric information (DNA, fingerprints, voice patterns, etc.), physical, physiological, and mental health condition, medical records and history, and sexual orientation.
4. Apart from a centrally enacted law, mental health sectoral regulatory frameworks are equally important to establish and concerning that, mental health professionals in India are accountable to the central and state mental health authorities under the Mental Healthcare Act 2017.^65^ Therefore, these sectoral authorities can supervise the kind of data obtained by digital interventionists and evaluate the extent of privacy protection.
5. India’s health laws should cover mental health and define privacy protection frameworks and continually update those to reflect an understanding of new and evolving risks by referring to established international standards.^48^^–^^50^^,^^54^
6. AI systems developers working in mental health should conduct a DPIA^50^ and refer to the IEEE Global Initiative on Ethics of Autonomous and Intelligent Systems.^51^^,^^52^^,^^54^
7. When considering the role of AI algorithms for supporting symptom monitoring or informing the diagnosis or care of mental health conditions, attention is necessary to avoiding harm to patients and accounting for risk of bias. Developers and researchers should be made aware of the possibilities of such biases due to the subjective and expressive nature of clinical data in text form as reported by mental health patients, and the inherent risks of associating mental disorders to certain patient groups or ethnicities.^66^ AI systems may reproduce biases in existing data,^67^ with potentially detrimental consequences to individuals. Also, poor quality data can adversely affect the use of AI systems^68^ and is further compounded in resource-constrained settings such as in India where there may be additional gaps, errors, or delays in data collection mechanisms. Accepted ethical principles such as autonomy, beneficence, and justice should be prioritized, particularly in the case of using data collected from patients from vulnerable groups who are susceptible to stigma and discrimination, such as many individuals seeking care for mental health challenges.^69^ Further, clinicians and therapists, due to lack of formal training in this space, may be unaware of managing granular data reported by an AI-based system or app, or may not feel completely confident with clinical insights gathered through these systems.^70^ To that end, efforts are equally needed by AI researchers to bridge the gaps in data and technology literacy for both patients and clinicians. The challenge herein is that there remain insufficient guidelines for the use of AI in health care settings,^71^^,^^72^ a challenge that is especially stark in lower-resource countries such as India. Even the NITI Aayog’s recommendations need further strengthening by adding dedicated guidelines on deploying AI research for patients with mental health disorders and other potentially stigmatizing conditions, in connection with point 6.
8. Caution is also needed due to the risk of perpetuating existing racial or ethnic biases or stigma with AI algorithms. A prominent study from the United States^73^ showed that an algorithm assigned the same level of risk of chronic diseases (i.e., hypertension, diabetes, renal failure, high cholesterol) to Black patients, who presented more risk factors and comorbidities than white patients. This racial bias reduced the number of Black patients identified as requiring additional medical care by more than 50%. The algorithm used health costs as a proxy indicator for health needs, which resulted in this bias. As less money was spent on Black patients who reported the same level of need, the algorithm falsely inferred that Black patients were healthier than white patients with the same medical problems. In the Indian context, there is a similar risk of exclusion of stigmatized groups. As part of the National Digital Health Mission, the Government of India has commenced the process of assigning a digital health ID to every citizen, which is voluntary “until all health data are mandatorily digitized.”^74^ As the digital health ID would offer the entire health data of an individual across providers and treatments (i.e., digital health profile) and given the risk of its potential linkage to the Aadhaar ID, there may be an unauthorized or unintended disclosure of an individual’s mental illness or other stigmatizing conditions (e.g., HIV, TB) resulting in the denial of access to crucial services or perpetuation of stigma. For example, a transgender individual may experience discrimination by an insurer or financial institution because they would have to reveal their gender and any prescription drugs or treatments taken.^74^ The linking of data across health care providers may accidentally worsen pre-existing social, cultural, and/or institutional stigma. Developers of algorithms under the National Digital Health Mission should be educated on these threats to users’ rights to access services. One example of improving algorithms is to avoid the use of convenient and seemingly effective proxy measures (e.g., health costs in the aforesaid U.S. study^73^) for ground truth, which could introduce bias.
9. We encourage investment and collaboration by mental health researchers and their technology partners to study and co-develop new mathematical models that can preserve privacy by limiting the information that one can obtain from released data, regardless of the extent of associated information.^54^ An example is “multi-party computation,” a “toolbox” of cryptographic techniques that allows joint computation of data by different partnering organizations working on a digital project, just as if they are sharing a database. Cryptographic techniques protect the data, so the involved parties can view relevant information of individuals, without their underlying sensitive data. This enables a secure analysis of data from different sources, which is pertinent in digital mental health interventions.^75^
10. Increasing awareness of data privacy among individuals with mental health conditions and their families is of paramount importance. People often tend to give consent to sharing their data, especially when interfacing with technology, which they would not have done had they known the purpose of providing such information. There is an urgent need for the inclusion of privacy rights and advisories in all digital mental health program material that is disseminated among beneficiaries, and at a deeper level, in the medical and technological training curricula to instill the fundamentals of privacy in medical and engineering graduates.^54^

**TABLE 4.** The Information Technology (Reasonable Security Practices and Procedures and Sensitive Personal Data or Information) Rules 2011: Rules 4, 5, 6, and 8

Salient Rules	Details
Rule 4	Organizations (referred to as the “body corporates”) seeking sensitive personal data should draft a privacy policy and make it easily accessible for individuals providing such data. The privacy policy should be clearly published on the website of the body corporate, and it should contain details on the type of information that is collected, its purpose, and the reasonable security practices that are undertaken to maintain the confidentiality of sensitive information.
Rule 5	(a) The body corporate should obtain consent from the person(s) providing information in writing/by fax/e-mail, before collecting sensitive personal data.
	(b) Information shall be collected only for lawful purposes, and it should be necessary for the purpose. It should be used only for its purpose, and shall not be retained for a period longer than required, for the purposes for which the information may lawfully be used, or is otherwise required under any other law for the time being in force.
	(c) The individuals providing sensitive data should be made aware of the fact that the information is being collected, its purposes and recipients, and the names and addresses of the agencies obtaining and retaining the information.
	(d) Offer the person(s) providing information an opportunity to review the information, and make corrections if required;
	(e) The body corporate should provide an option (before collecting the information) to the person(s) to not provide the information sought.
	(f) The body corporate should maintain the security of the information provided; and appoint a grievance officer, (with name and contact details on the website), responsible to address and resolve grievances of information providers over a maximum period of 1 month.
Rule 6	The body corporate must seek prior permission of the individual who provides sensitive data, before disclosing it to a third party, except if the request for such information is made by government agencies/third parties mandated under law or by a legal order.
Rule 8	International Standards (IS/ISO/IEC 27001) can be implemented by a body corporate to maintain data security. An audit of reasonable security practices and procedures should be conducted at least once a year or as and when the body corporate undertakes significant upgradation of its processes and computer resources.

While it is important to recognize that these 10 measures are not exhaustive, these guidelines can inform efforts to strengthen data protection frameworks and laws, including the existing draft of the Digital Information Security in Healthcare Act 2018 (DISHA) in India, which the Government of India plans to implement.^76^ DISHA includes provisions that regulate the generation, collection, access, storage, transmission, and usage of digital health data and the related personally identifiable information. Presently, DISHA includes the details of its regulated entities, affirmative rights of the individual providing sensitive data, guidelines on collection and processing of DHD, types of breach of DHD, and adjudication and enforcement in case of such offenses. Table 5 summarizes the Rights of the Data Subject under DISHA.^76^

**TABLE 5.** Rights of the Data Subject Under the Digital Information Security in Healthcare Act 2018 from the Ministry of Health and Family Welfare, Government of India

All Digital Health Data (DHD) is owned by the individual providing such data (the Owner), and her affirmative rights include:
1. The right to privacy, confidentiality, and security of this data.2. The right to give or refuse consent for the generation, collection, storage, transmission, access, or disclosure of this data. The owner may not be refused a health service if they exercise the right to refuse consent.3. The right to require the owner's explicit permission for each instance of transmission or use of their DHD.4. The right to access their DHD and the right to rectify inaccurate or incomplete DHD.5. The right to seek compensation for damages caused by a breach of DHD.

Linking of data across health care providers may accidentally worsen pre-existing social, cultural, and/or institutional stigma.

## IMPLICATIONS FOR OTHER COUNTRIES IN SOUTH ASIA

While the examples presented draw extensively from the case of data linkage with Aadhaar in India, these recommendations are relevant for many additional settings globally. Consideration of data safety in the context of emerging digital mental health interventions and expanding delivery of necessary care to those living with stigmatizing mental health conditions is relevant for many other lower-income countries, particularly among countries in the South Asian region where data safety policies are not yet well-established. In Table 6, we have illustrated the various contexts related to data protection in Bangladesh, Bhutan, Nepal, Pakistan, and Sri Lanka.

**TABLE 6.** Data Safety/Security Policies and Laws in South Asian Countries Adjoining India

Country	Data Safety Policies/Laws
Bangladesh	Concerning the newly enacted Mental Health Act in 2018, it has been critiqued that patient’s confidentiality and associated accountability of medical practitioners for failure to maintain confidentiality are not included in sufficient detail.^77^ Privacy laws are lacking; instead, there is a dependence on provisions within several other existing laws, or relevant sections in the country’s constitution such as Article 32 (protection of right to life and personal liberty), Article 39 (freedom of thought and conscience and of speech), and Article 43(b) (right to privacy for each citizen, of his correspondence and other means of communication).^78^ In December 2020, the government passed the Digital Security Rules, which call for organizations to establish “help desks” so that they could comply with the Digital Security Act 2018.^78^ As a consequence, employees can register complaints related to personal data misuse via these help desks. The Digital Security Act 2018 is inadequate to regulate a right as fundamental as data privacy, calling for new legislation. Requirements in GDPR may be difficult or costly to implement for many small companies in Bangladesh^78^; therefore, the proposed Personal Data Protection Bill in India serves as a reference,^78^^,^^79^ as it offers flexibility to smaller organizations.
Bhutan	Limited legislation related to mental health.^80^ The Information, Communications and Media Act of Bhutan 2018 includes data protection principles, which includes 7 of the 10 “second generation” principles of the 1995 European Union Data Protection Directive.^81^
Nepal	Privacy Act 2018 restricts processing of “sensitive data” in control of a public entity. Physical or mental health of a person are included as part of sensitive data, which can be processed “only during the diagnosis, treatment, and management of public health, and the delivery of health services to a person if such data has been made public by the concerned individual themselves.”^82^ Privacy Act has impacted the legal usage of “personal information” as it stipulates how “personal information” in public entities can be used, along with liabilities for breach.^81^
Pakistan	No specific law relating to data protection.^83^ In April 2020, the country’s Ministry of Information Technology and Telecommunication released a draft Personal Data Protection Bill for consultation before being presented to Parliament for debate. The Bill defines “sensitive personal data” as that which includes biometric data; information on the subject's physical, psychological, or mental health conditions as well as medical records, among other details. Sensitive personal data can be processed only with the explicit consent of the subject and only for defined purposes, such as: exercising any right or obligation conferred by law on the data controller in connection with the subject’s employment; protection of vital interests of the subject/another person; and where processing is undertaken for medical reasons/ by a health care professional.
Sri Lanka	The Personal Data Protection Bill is comprehensive^81^ covering both public and private sectors. The bill requires lawful grounds for processing users’ data and includes obligations of controllers and rights of users based on GDPR provisions. Key rights of GDPR are present, such as users’ “right to be forgotten” and protections against automated processing of data. The independence of the data protection authority, an independent public body authorized to supervise the application of the data protection law, provide expert advice on data protection issues, and handle complaints lodged against GDPR violations or relevant national laws, is not guaranteed.^81^ While mental health literacy has improved in Sri Lanka, the absence of consensus among stakeholders and legislative delays have hindered recent attempts to develop a new mental health act to replace the existing Mental Diseases Ordinance of 1956.^84^

Abbreviation: GDPR, General Data Protection Regulation.

These countries in the South Asian region account for more than 30% of adolescents globally^85^ while also experiencing a disproportionately greater share of the global burden of mental disorders.^86^ These challenges are compounded by having few mental health resources,^87^^,^^88^ highlighting the potential for digital interventions^89^^–^^91^ to bridge the care gap in the region. It should be noted that digital mental health interventions, particularly those involving online platforms and social media, could potentially lead to exposure of young users to hurtful content and hostile interactions with other users,^92^^,^^93^ threats to their data privacy,^94^^,^^95^ stigmatizing experiences that could impact their personal relationships, and unintended effects of online disclosure of personal information.^96^ Regulatory, systemic, and governmental efforts will be essential, with the participation of specialist and non-specialist health providers, technologists, and mental health interventionists to prioritize the protection of personal data and privacy of all individuals who receive these emerging interventions.

## CONCLUSIONS

In India, digital mental health practitioners and interventionists can refer to the guidelines outlined in this commentary and exercise substantial privacy protection while obtaining, storing, and using the personal data of individuals seeking care for mental health concerns. Regulatory agencies in this space should also consider the GDPR, DPIA, the IEEE Global Initiative on Ethics of Autonomous and Intelligent Systems, and NITI Aayog to further develop and refine their data protection efforts. Interventionists, who are obligated to adhere to these regulations, would then be enabled to conceive and develop privacy-sensitive intervention models. Data privacy policies are often complex and difficult to navigate, particularly for users with low literacy or those experiencing mental health symptoms; therefore, interventionists should clearly and succinctly communicate the kinds of data they would obtain from users.

Obtaining informed consent should mandatorily follow the privacy policy statement to ensure transparency rather than involve a checkbox indicating “agreement,” thus, giving the user ample opportunity to make an informed decision about their participation (which is often difficult due to the fast-paced nature of installing and using digital applications). Individuals refusing consent should be allowed to use the intervention, with their data excluded from outcome analysis. Provision of services should be de-linked with the receipt of individual personal data. A brief, clear, and comprehensive statement on the protection of personal data privacy, fully exercising “data minimization” and dissociation from Aadhaar would build greater trust and confidence in the digital intervention. This is particularly important as the digital mental health field continues to advance rapidly, where the implications of Aadhaar will require continued scrutiny to ensure the protection of the privacy, rights, and dignity of those living with mental health disorders.

## Notes

**Peer Reviewed**

**First published online:** September 8, 2021.
